# A novel serum m^7^G-harboring microRNA signature for cancer detection

**DOI:** 10.3389/fgene.2024.1270302

**Published:** 2024-02-07

**Authors:** Yaxin Chen, Yufang Xie, Liyun Bi, Hang Ci, Weimin Li, Dan Liu

**Affiliations:** ^1^ Institute of Respiratory Health, Frontiers Science Center for Disease-related Molecular Network, Department of Respiratory and Critical Care Medicine, West China Hospital, Sichuan University, Chengdu, Sichuan, China; ^2^ Jiujiang First People’s Hospital, Jiujiang, Jiangxi, China; ^3^ Center of Growth, Metabolism, and Aging, Key Laboratory of Bio-Resources and Eco-Environment, College of Life Sciences, Sichuan University, Chengdu, Sichuan, China

**Keywords:** m^7^G, microRNA, liquid biopsy, cancer diagnosis, pan-cancer

## Abstract

**Background:** Emerging evidence points to the exceptional importance and value of m^7^G alteration in the diagnosis and prognosis of cancers. Nonetheless, a biomarker for precise screening of various cancer types has not yet been developed based on serum m^7^G-harboring miRNAs.

**Methods:** A total of 20,702 serum samples, covering 12 cancer types and consisting of 7,768 cancer samples and 12,934 cancer-free samples were used in this study. A m^7^G target miRNA diagnostic signature (m^7^G-miRDS) was established through the least absolute shrinkage and selection operator (LASSO) analyses in a training dataset (*n* = 10,351), and validated in a validation dataset (*n* = 10,351).

**Results:** The m^7^G-miRDS model, a 12 m^7^G-target-miRNAs signature, demonstrated high accuracy and was qualified for cancer detection. In the training and validation cohort, the area under the curve (AUC) reached 0.974 (95% CI 0.971–0.977) and 0.972 (95% CI 0.969–0.975), respectively. The m^7^G-miRDS showed superior sensitivity in each cancer type and had a satisfactory AUC in identifying bladder cancer, lung cancer and esophageal cancer. Additionally, the diagnostic performance of m^7^G-miRDS was not interfered by the gender, age and benign disease.

**Conclusion:** Our results greatly extended the value of serum circulating miRNAs and m^7^G in cancer detection, and provided a new direction and strategy for the development of novel biomarkers with high accuracy, low cost and less invasiveness for mass cancer screening, such as ncRNA modification.

## Introduction

Early cancer detection is crucial for reducing cancer-caused mortality, prolonging patient survival and lessening the social burden (Bray et al., 2018). Due to the defects of high cost, invasiveness, poor compliance, and especially low accuracy, large-scale cancer screening is neither possible nor cost-effective based on present existing methods (So et al., 2021). A novel biomarker with greater effectiveness and less invasiveness for large-scale cancer screening is urgently needed due to the fact that early diagnosis of cancer could greatly extend patient survival.

N7-methylguanosine (m^7^G), a prevalent posttranscriptional modifications of RNA, is widely observed in both mRNAs and non-coding RNAs (e.g., tRNAs; micro-RNAs, miRNAs) (Luo et al., 2021; Wang et al., 2023), and plays essential roles in RNA metabolism, processing and function (Chen et al., 2022). m^7^G modification is dynamical and mainly regulated by the methyltransferase-like 1 (METTL1)/WD repeat domain 4 (WDR4) (Alexandrov et al., 2002), Williams–Beuren syndrome chromosome region 22 (WBSCR22)/tRNA methyltransferase activator subunit 11–2 (TRMT112) (Haag et al., 2015), and RNA guanine-7 methyltransferase (RNMT)/RNMT-activating miniprotein (RAM) (Trotman et al., 2017). Aberrant m^7^G modification was closely related to tumor occurrence and progression (Ma et al., 2021a; Luo et al., 2022). As the m^7^G methyltransferase of miRNAs, METTL1 exhibited hyperactivity in lung and bladder cancer, positively correlating with advanced clinical stages and high tumor grades (Pandolfini et al., 2019; Ying et al., 2021), while in colon cancer, it is significantly downregulated, actively participating in multiple tumor progression-related processes through its involvement in mediating miRNA maturation or in an m^7^G-tRNA codon-dependent manner (Ma et al., 2021b; Chen and Liu, 2021). These findings suggest that m^7^G plays a dual role in tumors and may unveil novel therapeutic targets for cancer patients.

miRNA is increasingly acknowledged as a valuable biomarker in various cancers. Recent studies have identified a subset of miRNAs with a modified m^7^G cap structure via different m^7^G sequencing techniques (Chen et al., 2022; Wang et al., 2023), such as miRNA let-7 (Chen and Liu, 2021) and miR-149-3p (Liu et al., 2019a). m^7^G can promote miRNA maturation or modulate miRNA binding to target genes, leading to alternations in the expression of their targets, including cancer-relevant genes. Previous studies have explored the prognostic value of m^7^G-related miRNAs and their association with immune infiltration in various cancer types (Yang et al., 2018; Hong et al., 2022; Zhang et al., 2022). In addition, Zhang et al. (2021) demonstrated the diagnostic performance of m^6^A target miRNAs in serum, with an area under the curve (AUC) of 0.979. Circulating miRNA, known for its highly stable as well as limited vulnerability to long-term storage at room temperature and freeze-thawing, is increasingly recognized as a valuable biomarker for cancer detection (Matsuzaki et al., 2021). However, the exploration of utilizing m^7^G target miRNAs (m^7^G-miRNAs) for cancer detection has not yet been undertaken.

Here, we utilized a total of 20,702 serum samples, covering 12 different cancer types and cancer-free controls, to establish a m^7^G-miRNAs diagnostic signature (m^7^G-miRDS) for cancer detection. The signature exhibited high accuracy in both the training and validation sets, with an AUC of 0.974 and 0.972, respectively. In addition, the m^7^G-miRDS showed excellent sensitivity in a particular cancer type, including bladder cancer, lung cancer and esophageal cancer. The diagnostic performance of m^7^G-miRDS was scarcely influenced by gender, age, or tumor stage. In summary, our study revealed the value of serum circulating m^7^G target miRNAs for cancer detection, and yielded a new path for developing novel biomarkers for large-scale cancer screening that are highly accurate, low cost and non-invasive for mass cancer screening, such as ncRNA modification.

## Materials and methods

### Dataset source

We retrieved serum miRNA expression data and corresponding clinical information from the NCBI Gene Expression Omnibus (GEO) database, applying the following inclusion criteria: 1) Samples from cancer groups were collected from treatment-naive patients with clear diagnoses of malignant tumors; 2) Samples from control groups were obtained from healthy volunteers or individuals with benign diseases; 3) No duplicate samples were included. In total, 20,702 samples from ten individual GEO datasets (GSE106817, GSE112264, GSE113486, GSE113740, GSE122497, GSE137140, GSE139031, GSE85589, GSE164174, GSE124158), were enrolled in this study (Supplementary Table S1). Those datasets included 12,934 cancer-free and 7,768 cancer samples across 12 cancer types, including bladder urothelial carcinoma (BLCA), breast invasive carcinoma (BRCA), colorectal cancer (CRC), esophageal carcinoma (ESCA), glioma (GBM), gastric cancer (GC), hepatocellular carcinoma (HCC), lung cancer (LC), pancreatic adenocarcinoma (PAAD), prostate adenocarcinoma (PRAD), sarcoma (SARC), and ovarian cancer (OV).

### Preprocessing of expression profiles

The Affymetrix datasets along with corresponding annotation files were downloaded individually. When the probes were converted to gene symbols, if multiple probes corresponded to the same gene symbol and their mean value was taken as the final value. All miRNA expression were integrated once every dataset was processed, normalized, and log2 transformed. To detect and remove the batch effect caused by non-biotechnological bias, we conducted batch correction utilizing the ComBat function from the R package sva (v.3.38.0) (Leek et al., 2012). In total, an expression matrix consisting of 20,702 samples and 2,525 miRNAs was utilized for further analysis.

### Functional annotation of m^7^G-harboring miRNAs

In this study, a total of 33 m^7^G target miRNAs were extracted from previously published studies (Liu et al., 2019b; Pandolfini et al., 2019) (Supplementary Table S2). To investigate the biological processes in which these miRNAs are involved, we initially predicted their potential targets using the miRWalk3.0 database (http://mirwalk.umm.uni-heidelberg.de/search_genes), with binding probability >95% and binding site position 3′UTR (Sticht et al., 2018). Only experimentally validated miRNA-target interactions from miRTarBase (https://mirtarbase.cuhk.edu.cn/), were considered for miRNA targets.

### Identification of differentially expressed m^7^G-harboring miRNAs

Differentially expressed m^7^G-harboring miRNAs between the cancer-free and cancer groups were identified using the R package limma (v3.10.3). The false discovery rate (FDR) was controlled using the Benjamini and Hochberg method. miRNAs with FDR <0.05 and |log2FC| >1, were considered significantly differentially expressed. Gene ontology (GO) enrichment and KEGG pathway enrichment analyses were performed to the biological functions and pathways associated with these genes using R package ClusterProfile (v3.6.0) (Yu et al., 2012).

### Construction of a diagnostic signature for cancer detection

To build a diagnostic signature for cancer detection, all enrolled samples (*N* = 20,702) were randomly equally divided into training and validation sets using the createDataPartition function of the R package caret (v6.0.88). Each group consisted of 10,351 samples, with 6,467 cancer-free controls and 3,884 cancers in each set. Details on those samples were presented in the Supplementary Table S3. Based on 22 differentially expressed m^7^G-harboring miRNAs (Supplementary Table S4), the least absolute shrinkage and selection operator (LASSO) analyses (100-fold cross-validation) was performed to determine the candidate m^7^G target miRNAs by using R package glmnet (v4.0.2). A diagnostic signature for cancer detection was established using 12 m^7^G target miRNAs (Supplementary Table S4). The model calculation formula is:

$$m^{7}G-\text{miRNA score}=\sum_{i=0}^{n}{Coef}_{i}*x_{i}$$

where Coef_i_ is the LASSO coefficient of each miRNA, and x _i_ is the expression level of each miRNA.

### Statistical analysis

All statistical analyses and picture drawing were implemented in R statistical software (v4.0.5). The diagnostic performance of the m^7^G-miRNAs signature was evaluated by receiver operating characteristic (ROC) curve analysis, including the area under the curve (AUC), sensitivity, specificity and accuracy. The relationship of m^7^G-miRNAs and each miRNA was determined using Spearman correlation analysis. Statistical significance between groups was calculated by a Wilcoxon test or Kruskal‒Wallis test at a confidence interval (CI) of 95%, with *p* < 0.05 considered statistically significant.

## Results

### Study design and data overview

**FIGURE 1:**
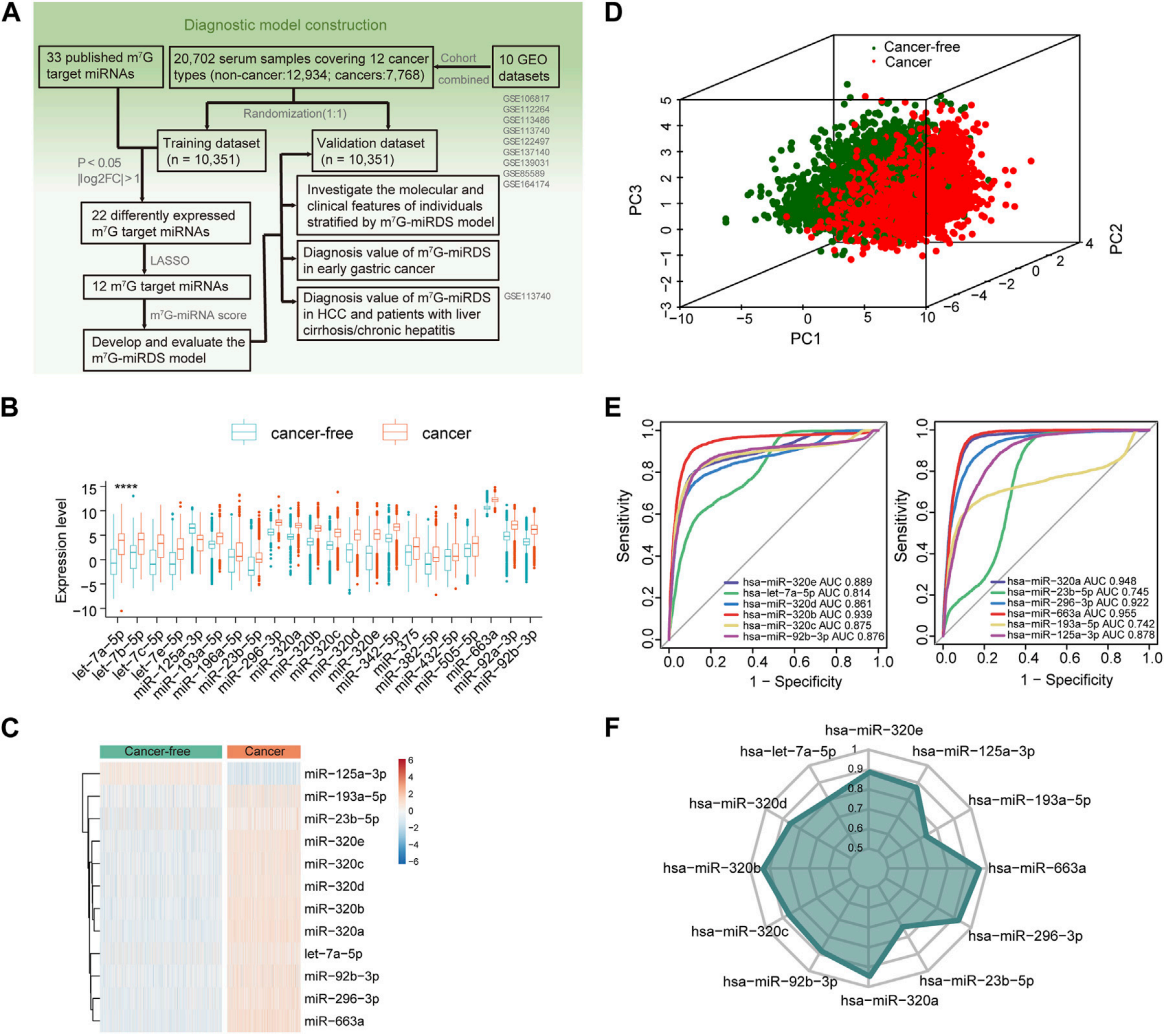
Identification of candidate m^7^G target miRNAs in serum. **(A)** Workflow illustrating the establishment of the serum m^7^G-miRNAs signature for cancer detection. **(B)** Boxplot showing 22 differentially expressed m^7^G target miRNAs between the cancer-free and cancer groups, according to the criteria of FDR <0.05 and log2|fold change| > 1. Statistical significance values correspond to *p* values as follows: **** <0.0001. **(C)** Heatmap of the expression of 12 candidate m^7^G target miRNAs identified by LASSO in the cancer-free and cancer groups. **(D)** Principal component analysis between cancer-free and cancer groups based on the 12 candidate m^7^G target miRNAs. **(E)** ROC curve showing the performance of each candidate m^7^G target miRNA individually detecting cancer patients in the training set. The area under curve (AUC) of a single miRNA ranged from 0.742 to 0.955. **(F)** Radar chart showing the AUC of each candidate m^7^G target miRNA individually detecting cancer patients in the validation set.

A schematic diagram of the study design was presented in Figure 1A. In total, an integrative dataset of 20,702 serum samples and 33 m^7^G target miRNAs was utilized to build a m^7^G target microRNAs diagnostic signature (m^7^G-miRDS) for cancer detection. In the training and validation cohort, the average age of participants in the both was 62.5 years, and the proportion of female was 30.7% and 31.3%, respectively.

GO and KEGG enrichment analysis were performed to explore the biological processes regulated by m^7^G target miRNAs. The results underscored the predominant enrichment of these genes in pathways related to cancer and RNA metabolism, including TGF-β receptor signaling pathway, RNA metabolism process and RNA localization (Supplementary Figures S1D–SE; Supplementary Table S5).

### Identification of candidate m^7^G-harboring miRNAs in serum

Utilizing expression profiles of 33 pan-cancer datasets from The Cancer Genome Atlas (TCGA) project, we observed aberrant expression of METTL1 and WDR4, known as m^7^G methyltransferase, across multiple cancer types. Specially, the two genes were downregulated in colon adenocarcinoma compared to controls, while upregulated in diffuse large B-cell lymphoma and thymoma. In addition, they demonstrated significant independent prognostic value in cancers such as BLCA, ESCA and OV, as illustrated in Supplementary Figures S1A-C. These findings implied that m^7^G dysregulation was closely related to tumor occurrence and progression.

In the training set, 22 of 33 m^7^G target miRNAs were significantly differentially expressed when comparing the cancer group with the cancer-free controls (FDR<0.05 and log_2_|fold change| > 1). Among these, 21miRNAs exhibited significant upregulation in cancers, and only miR-125a-3p was significantly downregulated (Figure 1B and Supplementary Table S4). Then, we identified a total of 12 candidate m^7^G target miRNAs using the LASSO (Supplementary Figures S1F,G). Supplementary Table S4 listed 12 m^7^G target miRNAs and their corresponding coefficients. Based on the expression profiles of the 12 candidate miRNAs, unsupervised hierarchical clustering demonstrated a distinct separation between cancers and cancer-free controls (Figure 1C). This observation was further supported by principal component analysis (Figure 1D). These results underscored significant differences in expression patterns between two groups, providing valuable insights for the development of a diagnostic signature for cancer detection.

### Estimation for diagnostic performance of each candidate miRNA

The diagnostic performance of each candidate miRNA was assessed individually for cancer detection. Each miRNA exhibited a significant diagnostic capability in distinguishing cancers from cancer-free controls, with AUC values ranging from 0.742 for miR-193a-5p to 0.955 for miR-663a (Figure 1E). The similar finding was also observed in the validation cohort (Figure 1F). Those results suggested that identified candidate m^7^G target miRNAs hold promise as biomarkers for cancer detection.

### Construction of a diagnostic signature for cancer detection

**FIGURE 2:**
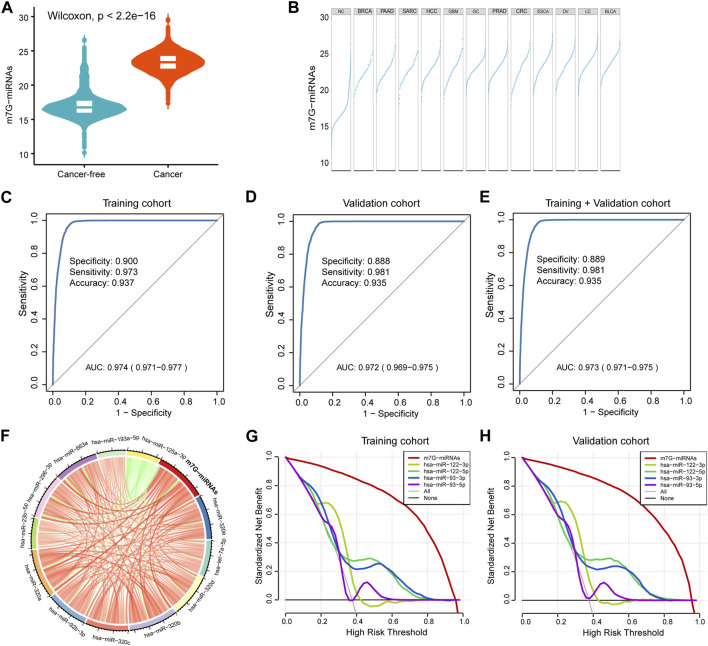
Construction of a serum m^7^G target miRNA signature for cancer detection. **(A)** Violin plot showing differences in m^7^G-miRNAs scores between the cancer-free and cancer groups. **(B)** Plot displaying differences in m^7^G-miRNAs scores across different cancer types. **(C–E)** The ROC curve showing the diagnostic performance of m^7^G-RDS in the training set **(C)**, validation set **(D)** and entire set **(E)**. **(F)** Circle plot depicting the relationship between the m^7^G-miRNA score and the expression of each miRNA using Spearman statistical analysis. Green represents a negative correlation, and red represents a positive correlation. **(G,H)** The decision curve showing differences in decision threshold probability between m^7^G-RDS and other serum biomarkers in the training set **(G)** and validation set **(H)**.

Utilizing the 12 identified m^7^G target miRNAs, we built a diagnostic signature for cancer detection, named m^7^G-miRDS. The m^7^G-miRNAs score in cancers was significantly higher than that in cancer-free controls (Figure 2A). The distribution of m^7^G-miRNA scores in each cancer type showed that BRCA patients had the lowest median m^7^G-miRNA scores, while BLCA patients had the highest median m^7^G-miRNA scores (Figure 2B). The m^7^G-miRDS model, a 12-m^7^G-miRNAs set, showed a higher diagnostic power than each candidate miRNA alone in distinguishing cancer samples from cancer-free controls in the training cohort, with an AUC and 95% confidence interval (CI) of 0.974 (0.971–0.977) (Figure 2C). Similar to the training cohort, the m^7^G-miRDS model also showed a high diagnostic performance in the validation cohort, with an AUC of 0.972% and 95% CI, 0.969 to 0.975 (Figure 2D). Additionally, the m^7^G-miRDS demonstrated a satisfactory diagnostic value in the entire set (Figure 2E).

By Spearman correlation analysis, we observed a remarkable positive correlation between m^7^G-miRNAs and 12 miRNAs, except hsa-miR-125a-3p (Figure 2F and Supplementary Table S6). Previously published studies reported the important value of hsa-miR-93 and hsa-miR-122 in the diagnosis and prognosis of various cancer types (Luna et al., 2017; Gao et al., 2019; Faramin Lashkarian et al., 2023). In decision curve analyses, m^7^G-miRNAs demonstrated an absolute superiority net benefit within a wide range of decision-making threshold probabilities compared to hsa-miR-93 and hsa-miR-122 neither in training set not validation set (Figures 2G,H).

### Independence and accuracy of the m^7^G-miRDS

**FIGURE 3:**
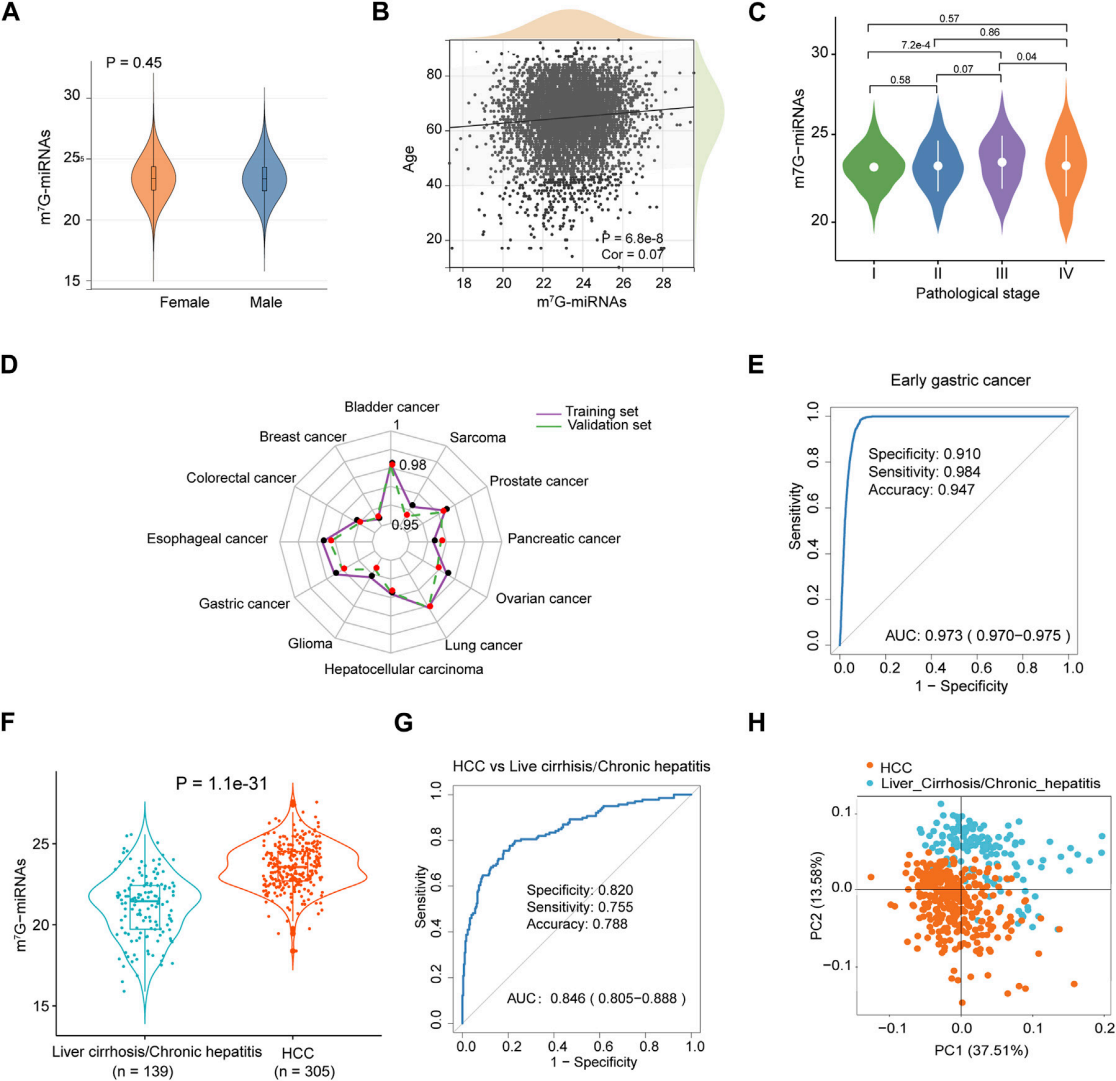
Diagnostic performance of m^7^G-RDS in different clinical features and cancer types. **(A)** Differences in m^7^G-miRNAs scores between female and male individuals. **(B)** Correlation between m^7^G-miRNAs score and age through Spearman analysis. **(C)** Violin plot illustrating differences in m^7^G-miRNAs scores among different stages. **(D)** The radar chart showing the diagnostic ability of m^7^G-RDS in each cancer type of the training set (purple polyline) and the validation set (green polyline). **(E)** The ROC curve showing the diagnostic value of m^7^G-RDS in early gastric cancer. **(F)** Plot of differences in m^7^G-RDS between patients with hepatocellular carcinoma (HCC) and patients with liver cirrhosis or chronic hepatitis. **(G)** ROC curve showing the ability of m^7^G-RDS to distinguish HCC patients from liver cirrhosis/chronic hepatitis patients. **(H)** Principal component analysis for the 12 candidate m^7^G target miRNAs in HCC and liver cirrhosis/chronic hepatitis patients.

Considering the effect of patient clinicopathological characteristics on diagnostic efficacy, such as gender, age and pathological stage, we tested the diagnostic performance of m^7^G-miRDS stratified by patient gender. No significant difference in m^7^G-miRNA scores was observed between female and male patients (*p* = 0.45, Figure 3A). Similar to gender, we also did not observe a significant correlation between m^7^G-miRNA scores and patient age (*R* = 0.07, Figure 3B). According to the subgroups classified by tumor stage, there was no significant difference in the m^7^G-miRNA scores between different pathological stages, except for stage I and stage III (Figure 3C). In conclusion, m^7^G-miRDS was not interfered by the gender and age of patients, underscoring its robustness as a biomarker for distinguishing cancers from controls.

### Diagnostic performance of m^7^G-miRDS in different cancer types

To evaluate the discriminative ability of m^7^G-miRNAs across various cancer types, we mixed each cancer type separately with cancer-free controls. As expected, m^7^G-miRDS showed superior discriminatory capability, with the highest AUC and 95% CI for bladder cancer in the training and validation cohort (0.982, 0.979–0.986 and 0.981, 0.978–0.985, respectively), and the lowest AUC and 95% CI for breast cancer in both sets (0.954, 0.946–0.963 and 0.955, 0.947–0.963, respectively) (Figure 3D and Supplementary Table S7). Despite a slight diminish in diagnostic ability of m^7^G-miRDS for individual cancer type, it maintained very high sensitivity. The results suggested that m^7^G-miRDS could identify over 92% of patients with a given cancer type, resulting in a lower incidence of missed diagnosis.

### Utility of m^7^G-miRDS in diagnosing early cancer and non-tumor diseases

Among ten datasets, the dataset GSE164174 included samples derived from early gastric cancer, and the training and validation cohort consisted of 698 cancer-free controls and 711 samples from patients with early gastric cancer, with 705 stage I and 5 stage II samples. In Figure 3E, the m^7^G-miRDS demonstrated promising performance in diagnosing early gastric cancer, with an AUC of 0.973 (95% CI, 0.970–0.975), a specificity of 0.910, a sensitivity of 0.984 and an accuracy of 0.947.

In addition, the dataset GSE113740 included 46 chronic hepatitis and 93 liver cirrhosis serum samples, thus we also investigated the capability of m^7^G-miRDS in distinguishing between cancer and non-tumor diseases. The model exhibited a remarkable accuracy in discriminating HCC individuals from patients with chronic hepatitis or liver cirrhosis (Figures 3F–H), with an AUC of 0.846 (95% CI: 0.805–0.888), surpassing the performance of traditional biomarkers, such as alpha-fetoprotein (AUC, 0.65; specificity, 51.4%; sensitivity, 73.3%) (Yamamoto et al., 2020). The above results indicated that chronic disorders might not significantly affect the diagnostic performance of m^7^G-miRDS.

## Discussion

Growing evidence indicates a critical role of m^7^G in the development of human disease, especially cancer (Wang et al., 2023; Xia et al., 2023). The m^7^G modification can occur in miRNAs, and aberrant m^7^G levels are closely associated with tumorigenesis and progression via regulation of the expression of downstream targets, such as multiple oncogenes and tumor suppressor genes (Ma et al., 2021a; Luo et al., 2022). miRNAs derived from blood have recognized as valuable biomarker for the diagnosis and prognosis of cancer, owing to their noninvasive and cost-effective nature (Bagnoli et al., 2016; Hussen et al., 2021). However, utilizing a specific set of m^7^G target miRNAs as a diagnostic signature and its relationship with cancer detection largely unexplored. Here, utilizing a total of 20,702 serum samples across 12 distinct cancer types, we identified a novel 12-m^7^G-target-miRNA biosignature for cancer detection. The m^7^G-miRDS demonstrated effective discrimination between cancers from cancer-free controls, independent of patient gender and age.

There have been studies focusing on the development and validation of signatures for cancer prognosis or diagnosis by utilizing m^7^G regulators (e.g., m^7^G methyltransferase) or RNA modification related miRNAs (e.g., m^6^A target miRNAs), demonstrating high specificity and sensitivity (Zhang et al., 2021; Hong et al., 2022; Li et al., 2022; Ying et al., 2023). Early detection of cancer leads to better treatment and increased survival rates (Crosby et al., 2022). However, existing studies has not extensively explored the relationship between m^7^G and cancer detection, especially concerning the diagnostic performance of m^7^G target miRNAs in serum. In this study, a set of 12 target miRNAs, namely, miR-320e, let-7a-5p, miR-320a, miR-320d, miR-320b, miR-320c, miR-92b-3p, miR-23b-5p, miR-296-3p, miR-663a, miR-193a-5p, and miR-125a-3p, was identified as a diagnostic signature to discriminate cancer from cancer-free controls. Among these 12 m^7^G-miRNAs, four belong to the miR-320 family, known for anti-oncogene target miRNA for cancer therapy (Liang et al., 2021). Notably, miR-320b and miR-320d were identified as prediction biomarkers for platinum resistance in ovarian cancer patients (Liu et al., 2022). Aberrant expression of miR-320e, let-7a-5p and miR-92b-3p have been acknowledged as a prognostic biomarker in colorectal cancer (Perez-Carbonell et al., 2015; Pichler et al., 2017; Bustos et al., 2021). Additionally, we discovered that miR-320b and miR-320e, which can be modified by m^6^A and m^7^G, were identified as diagnostic signatures for cancer detection in both Zhang et al., 2021 study and our research. Those findings enhance our understanding of the molecular intricacies involving miRNAs as well as their RNA modifications, presenting a new avenue for cancer detection via using distinct specific biosignatures, especially modified non-coding RNAs.

However, our study has a number of drawbacks: 1) Due to the lack of corresponding stage information except gastric cancer, the performance of m^7^G-miRDS in diagnosing other cancers with early stages still needs to be further investigated. 2) The function of several m^7^G target miRNAs still requires further validation through low-throughput experiments. 3) There aren't enough m^7^G-RIP-seq data from the serum of cancer patients, and more m^7^G target miRNAs could be candidates for the development of a signature. Together, these results suggest a diverse potential of m^7^A target miRNAs in cancer detection and the creation of cutting-edge therapeutic approaches.

## Conclusion

A 12 m^7^G-miRNAs biosignature to diagnose cancer was identified. Our results suggest that the importance of serum circulating m^7^G target miRNAs in cancer, and further large prospective cohort studies are warranted to validate our findings.

## Data Availability

All serum miRNA expression profiles with corresponding clinical information were available in GEO (https://www.ncbi.nlm.nih.gov/geo/), and the related accession number was provided in the supplementary materials.

## Supplementary material

SUPPLEMENTARY FIGURE S1 The expression level and survival analysis of m^7^G methyltransferase and the function annotation of m^7^G target miRNAs. **(A)** Heatmap illustrating the prognosis implications of METTL1 and WDR4 genes across 33 cancer types. **(B,C)**. Differential expression analysis of the METTL1 (B) and WDR4 (C) genes in TCGA pan-cancer cohort. **(D,E)** Barplot displaying functional annotation of the included 33 m^7^G target miRNAs using gene ontology (GO) **(D)** and KEGG pathway enrichment analysis **(E)**. **(F)** The tuning parameters (log(lambda)) of m^7^G target miRNAs were selected to cross-verify the error curve. According to the minimal criterion and 1-se criterion, perpendicular imaginary lines were drawn at the optimal value. **(G)** The LASSO coefficient profile of 12 candidate m^7^G target miRNAs and perpendicular imaginary lines were drawn at the value chosen by 100-fold cross-validation.

SUPPLEMENTARY TABLE S1 Ten GEO datasets used in this study and corresponding sample sizes and clinical features of each dataset.

SUPPLEMENTARY TABLE S2 A list of m^7^G-harboring miRNAs derived from previously published studies. Functional annotation for 33 m^7^G target miRNAs. A list of. List of 12 m^7^G target miRNA obtained by using the LASSO method.

SUPPLEMENTARY TABLE S3 Details on samples in the training set and validation set.

SUPPLEMENTARY TABLE S4 A list of significantly differentially expressed m^7^G target miRNAs (FDR < 0.05 and |log2(FC)| > 1) and 12 candidate m^7^G-miRNAs.

SUPPLEMENTARY TABLE S5 Functional annotation for 33 m^7^G target miRNAs.

SUPPLEMENTARY TABLE S6 Spearman correlation analysis between m^7^G-miRNAs and 12 miRNAs.

SUPPLEMENTARY TABLE S7 Summary of AUC, sensitivity, specificity and accuracy of m^7^G-miRDS for each cancer type in the training and validation cohorts. AUC: area under the curve.
