# Supplementary figures and images for: A novel serum m7G-harboring microRNA signature for cancer detection
Source: Front Genet. 2024 Feb 7;15:1270302. doi: 10.3389/fgene.2024.1270302 (PMC10879580; doi:10.3389/fgene.2024.1270302)

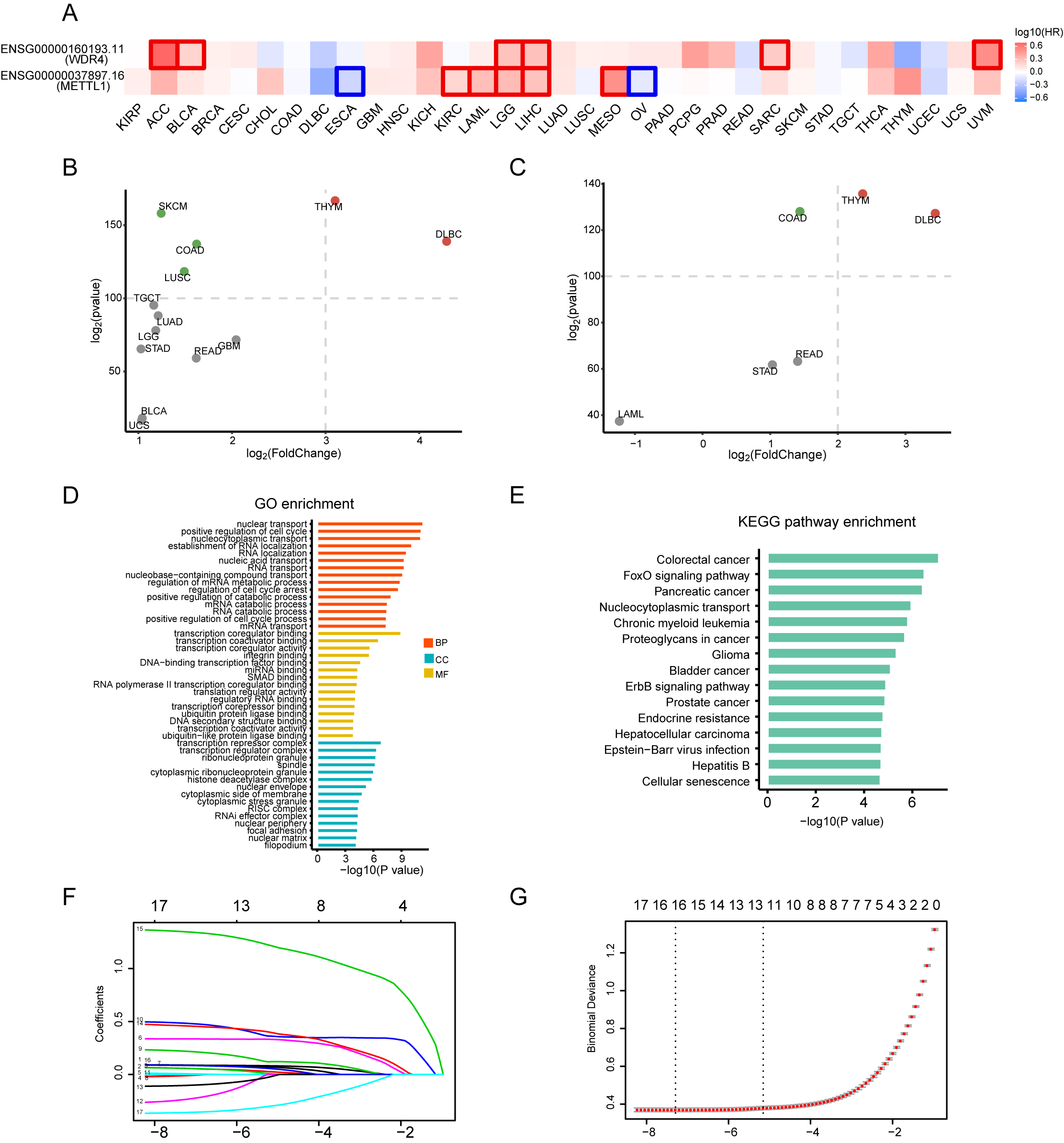

Supplement: Supplementary file 3 [file Image1.TIF]
